# Circulating Tumor DNA Methylation Biomarkers for Characterization and Determination of the Cancer Origin in Malignant Liver Tumors

**DOI:** 10.3390/cancers15030859

**Published:** 2023-01-30

**Authors:** Tina Draškovič, Nina Zidar, Nina Hauptman

**Affiliations:** Institute of Pathology, Faculty of Medicine, University of Ljubljana, 1000 Ljubljana, Slovenia

**Keywords:** malignant liver tumors, primary malignant liver tumors, liver metastases, liquid biopsy, cell-free DNA, circulating tumor DNA, tissue of origin determination, DNA methylation

## Abstract

**Simple Summary:**

Malignant liver tumors consist of primary tumors and metastases. A correct diagnosis can be achieved with different methods; one of them is the analysis of the cell-free DNA methylation profile in liquid biopsy, which allows for the detection, characterization, and determination of cancer origin. Our review aims to provide an insight into methylation changes in circulating tumor DNA from patients with different malignant liver tumors and may serve as a starting point for further research.

**Abstract:**

Malignant liver tumors include primary malignant liver tumors and liver metastases. They are among the most common malignancies worldwide. The disease has a poor prognosis and poor overall survival, especially with liver metastases. Therefore, early detection and differentiation between malignant liver tumors are critical for patient treatment selection. The detection of cancer and the prediction of its origin is possible with a DNA methylation profile of the tumor DNA compared to that of normal cells, which reflects tissue differentiation and malignant transformation. New technologies enable the characterization of the tumor methylome in circulating tumor DNA (ctDNA), providing a variety of new ctDNA methylation biomarkers, which can provide additional information to clinical decision-making. Our review of the literature provides insight into methylation changes in ctDNA from patients with common malignant liver tumors and can serve as a starting point for further research.

## 1. Introduction

Malignant liver tumors are one of the most common malignancies with poor prognoses and a low overall survival rate [1]. They consist of primary malignant tumors and metastases of other primary tumors that can spread to the liver [2,3]. The most prevalent primary malignant liver tumor is hepatocellular carcinoma (HCC), followed by intrahepatic cholangiocarcinoma (CCA) [1,2].

The incidence among malignant liver tumors is higher for liver metastases than for primary malignancies [4]. Approximately 25% of solid organ metastases are found in the liver, making it one of the most common metastatic sites [5]. Recent data demonstrate that approximately 5.1% of patients have liver metastases at the time of primary cancer diagnosis [6]. Liver metastases may develop because of the dual blood supply to the liver through the systemic and portal circulations as described in the “mechanical or hemodynamic hypothesis,” or some primary tumors may selectively target the liver as described in the “seed-and-soil hypothesis” [7,8]. The second mechanism is based on the assumption that the hepatic sinusoidal epithelium facilitates the invasion of metastatic cells into the liver parenchyma due to fenestrations [8].

Malignant tumors that can metastasize to the liver include carcinomas, melanomas, lymphomas, sarcomas, and germ cell tumors [4,9]. The most prevalent liver metastases are carcinomas (92%), with the most common subtype being adenocarcinoma (75%), followed by neuroendocrine carcinoma and squamous cell carcinoma [9]. The major sources of liver metastases are colorectal carcinomas, followed by carcinomas of the pancreas, breast, lung, and stomach. Metastases from melanoma, ovarian, endometrial, esophagus, small intestine, prostate, and renal primary carcinomas also occur (each with an incidence of less than 4%) [3,9]. Sometimes, the primary site of a metastatic tumor cannot be determined, referred to as carcinoma of unknown primary (CUP) [9]. The data suggest that the majority of CUPs are found in the liver (between 24% and 50%). These patients have a higher mortality rate than patients without liver metastases. CUP often has similar or preserved biological and molecular characteristics of the tumor from which it originates [10,11,12].

Differentiation between adenocarcinoma and HCC is usually possible on the basis of morphologic and immunohistochemical features. However, in some cases, HCC forms acinar structures or is poorly differentiated and indistinguishable from metastatic adenocarcinoma [4]. Various factors, including the etiology of liver disease, the rate of cancer progression, as well as molecular heterogeneity between different metastatic samples and samples within the same tumor mass, complicate the characterization of malignant liver tumors. However, because of different prognoses and treatment options, the differentiation and detection of the primary tumor site or tissue of origin (TOO) are of vital importance [1,9,13,14].

Patients with liver metastases have worse overall survival than patients with metastases at other sites [15,16,17]. The early detection and accurate classification of liver metastases are crucial for implementing effective and tailored treatment approaches according to the extent of liver disease, biology, and TOO [3]. The study of molecular mechanisms underlying liver metastatic behavior and the identification of novel diagnostic and prognostic biomarkers to assess the risk of liver metastases are of great importance.

Recently, a variety of new methylation biomarkers has emerged to help characterize and determine the origin of cancer. In this review, we will discuss and provide insights into methylation in cancer, ctDNA, the role of methylation in determining the TOO, and methylation changes in the ctDNA of patients with the most common primary malignant liver tumors and liver metastases.

## 2. Methylation in Cancer

Recent genetic analyses of cancer progression have failed to identify the specific driver mutations of carcinogenesis, denying the assumption that mutations alone contribute to cancer development. Emerging evidence suggests that metastatic features may result from epigenetically regulated tumor cell gene expression [18,19].

DNA methylation is one of the epigenetic mechanisms that regulate gene expression in eukaryotes. By covalently adding a methyl group to the 5′ positions of a cytosine, a 5-methylcytosine (5-mC) forms, typically within the CpG islands of promoter regions. This regulatory mechanism plays an important role in ensuring a stable gene expression profile, embryonic development, the maintenance of cell identity, tissue differentiation, and genome stability [20,21,22]. Therefore, it is not surprising that changes in methylation play an important role in cancer development. A combination of epigenetic events can alter the expression pattern of oncogenes or tumor suppressor genes, contributing to the development of a tumor phenotype [22,23]. Epigenetic changes that are considered early events in carcinogenesis may be suitable for early cancer detection as DNA methylation biomarkers [20,24,25,26]. Recent results further support the idea that the hypermethylation of otherwise methylation-resistant sites is established in the early stages of cancer development and remains methylated in advanced stages [25].

Alterations in the methylation pattern are highly pervasive across a particular tumor type. The unique methylation profile of cells can be used to distinguish cancer cells from healthy tissue and by identifying the TOO of the DNA [27,28]. Moreover, the methylation pattern can be used as a potential marker in the search for the origin of metastases, including CUP [28,29]. Epigenetic profiles inherently reflect differences between the normal and malignant tissues, which contributes to cancer detection and TOO determination [30]. Shen et al. showed that machine learning-derived classifiers based on differentially methylated regions could detect early- and late-stage cancer with high accuracy, sensitivity, and specificity [31]. Furthermore, Moss et al. showed that even an analysis of a small number of methylated loci could reliably discriminate between different cancer types [28]. A prospective case–control sub-study also supported the high significance of methylation patterns, which could detect more than 50 cancers at all stages [30]. Therefore, the altered or unaltered genetic and epigenetic landscape of metastases, when compared to primary cancer, may play a significant role not only in diagnostics but also in cancer monitoring, prognosis, treatment, influencing drug efficacy, or promoting the development of drug resistance [32].

Another intriguing class of methylation biomarkers associated with carcinogenesis is 5-hydroxymethylcytosine (5 hmC). In a recent study, disease-specific changes in 5 hmC successfully distinguished HCC from other primary and metastatic cancers [33]. This study points to the diagnostic potential of 5 hmC signatures in cell-free DNA (cfDNA) for cancer characterization and stage prediction.

## 3. Circulating Tumor DNA

Over the last decade, liquid biopsy has attracted considerable attention in oncology diagnostics [34,35]. In contrast to tissue biopsy, several advantages contribute to the widespread use of liquid biopsy: it is a simple, inexpensive, rapid, minimally invasive, easily accessible, and patient-friendly biological sampling procedure. It provides information on the longitudinal dynamics of tumor biomarkers and reflects the current complexity of the patient’s total tumor mass [36]. CfDNA is a mixture of small DNA fragments that circulate freely in the bloodstream. It originates from dying cells and the spontaneous leaching of DNA from living cells, thereby showing the body’s present state, including any malignancy [37]. A part of cfDNA is ctDNA, which is the most studied specimen in liquid biopsy in cancer. Viable apoptotic and necrotic tumor cells in the tumor microenvironment are the major source of ctDNA. It can account for a substantial proportion of cfDNA ranging from 3% to 93%, depending on the tumor location, size, and vascularization [38]. Additionally, the concentration of ctDNA may be affected by hepatic and renal clearance as well as by antitumor treatments. Large-sized tumors generally produce more ctDNA than small-sized ones, although tumor types also play a role. Patients with advanced disease have higher ctDNA levels than patients with localized disease, in whom blood levels actually increase with the increasing tumor stage [39]. A recent study suggests that mutation fingerprints and methylation patterns of ctDNA differ from the non-tumorous fraction of cfDNA [40]. Aberrations such as point mutations, copy number variation, microsatellite changes, and methylation changes can be detected in the ctDNA of most cancer patients [19,41,42]. Many studies have analyzed potential biomarkers from both tissue samples and cfDNA, demonstrating the persistence of aberrations in both samples [40,43,44,45,46]. These findings suggest that cfDNA from the plasma is comparable to conventional invasive tissue biopsies.

## 4. Role of Methylation in the Tissue-of-Origin Determination

A relatively high percentage of CUP among malignant liver tumors illustrates that the distinction between liver metastases, primary malignant liver tumors, and the TOO determination can be challenging. When TOO is discovered during the course of treatments, a metastasis of known origin (formerly CUP) generally responds well to the optimized treatment [47]. By determining the cancer type and TOO, unnecessary diagnostic tests can be avoided. In tissue samples, epigenetic profiling accurately predicted a primary cancer origin in 87% of the 216 CUP patients analyzed [41]. Similar to tissue samples, epigenetic profiling in cfDNA is a promising tool for TOO identification [28]. Namely, several research groups have demonstrated the utility of different epigenetic profiling in cfDNA for classification and TOO determination [42,48,49].

A comprehensive targeted methylation sequencing of 9223 hypermethylated CpG sites in the cfDNA of patients with various advanced cancer types was performed. This study successfully demonstrated the utility of median methylation scores and cancer-type-specific methylation signatures for cancer detection, classification, and differentiation between colorectal cancer (CRC), breast cancer, melanoma, and non-small cell lung cancer (NSCLC). The TOO determination was accurate in 83.5% of cases for 32 common cancer types [49].

The Circulating Cell-free Genome Atlas Study (CCGA), one of the largest blood-based clinical trials to date, investigated the suitability of genome-wide cfDNA sequencing combined with machine learning for cancer detection and TOO determination. It was designed as a prospective, multicenter case–control observational study with longitudinal follow-up. All sub-studies included 15,245 participants with more than 50 cancers in metastatic and non-metastatic stages. The first CCGA sub-study found that whole-genome bisulfite sequencing, which investigates genome-wide methylation patterns, was superior to whole-genome sequencing and targeted sequencing approaches, which investigate the copy number and single nucleotide variants or small insertions and deletions [42]. The second case–control sub-study evaluated the performance of the targeted methylation analysis of cfDNA (including >100,000 distinct CpG regions) for the detection and localization of multiple cancers at all stages. The TOO was predicted in 96% of samples with a 93% accuracy. The detection ability varies by cancer type and increases significantly with the increasing disease stage, from 18% in stage I to 93% in stage IV [48]. Because this study also included all cancer types covered in this review, it shows the potential of using methylation signatures in cfDNA for classification and TOO determination.

## 5. Methylation Signature of cfDNA in Malignant Liver Tumors

The DNA from normal liver cells represents about 1% of the total cfDNA in healthy individuals and signifies the largest portion of non-hematopoietic cfDNA [27,28,50]. Unsurprisingly, the hepatic portion of cfDNA increases in patients with various liver diseases, which also correlates with elevated liver enzymes in the blood [27]. A significant increase in the total cfDNA concentration is observed in cancer patients, especially in patients at later stages of the disease and with metastases [51,52]. However, the presence of ctDNA was also detected in the early stages of cancer [39].

**Figure 1:**
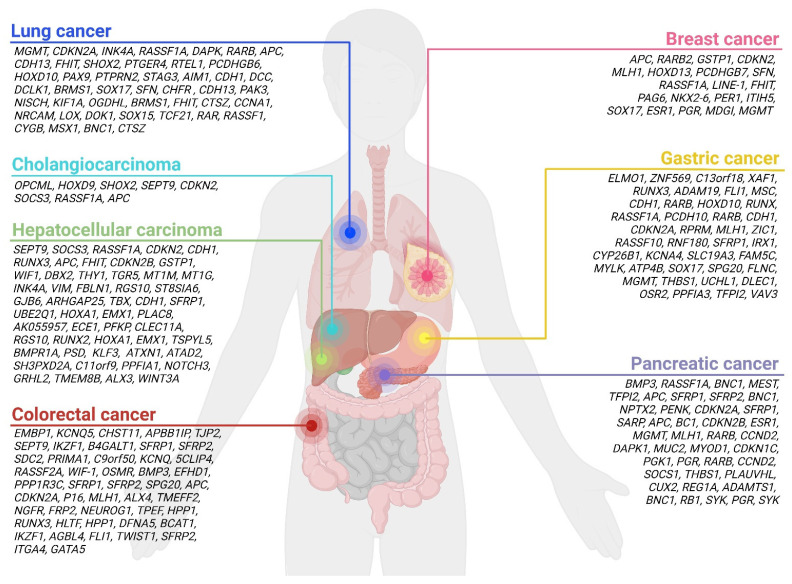
Overview of aberrantly methylated genes detected in patients with the most common primary malignant liver tumors and malignant tumors that most commonly metastasize to the liver. Created with BioRender.com.

A systematic mapping review shows that most studies and publications in the field of cfDNA focus on the most common cancer types [53]. These include the most common malignant primary liver tumor, HCC, and the most common liver metastases, such as CRC, lung, and pancreatic cancer metastases [53]. Therefore, this review focuses on the utility of cfDNA methylation in patients with the most common primary malignant liver tumors and liver metastases, as well as their ability to differentiate (Figure 1).

### 5.1. Most Common Primary Malignant Liver Tumors

#### 5.1.1. Hepatocellular Carcinoma

HCC is the most common primary malignancy of the liver and can usually be distinguished from metastatic tumors by its histopathologic and immunophenotypic features [54,55,56]. The discovery of differentially methylated regions (DMR) has enabled the identification of many genes involved in hepatocarcinogenesis (Table 1).

A meta-analysis of 2019 publications on DNA methylation markers in HCC identified six aberrantly methylated genes (*RASSF1A*, *PCDKN2A*, *CDH1*, *RUNX3*, *GSTP1,* and *WIF1*) in the serum of HCC patients compared to the serum of healthy individuals [45]. Additionally, the hypermethylation of specific methylation sites in HCC was shown to occur early in cancer development and remains in advanced stages [25].

Kisiel et al. performed pilot phase I and phase II studies to investigate the detection of HCC with methylated DNA biomarkers in the plasma. A 6-marker panel (*HOXA1*, *EMX1*, *AK055957*, *ECE1*, *PFKP,* and *CLEC11A* normalized by the *B3GALT6*) detected HCC with a 95% sensitivity and a 92% specificity. The detection rate increased with the disease stage and outperformed alpha-fetoprotein detection [44]. This study proved that HCC could be accurately detected by blood tests. Wen and colleagues developed a novel high-throughput DNA methylation method MCTA-Seq (methylated CpG tandems amplification and sequencing), which analyzed thousands of CpG islands in a single liquid biopsy sample. Four differentially methylated genes in the cfDNA of HCC patients were identified using this method. The sensitivity and specificity of HCC detection in cfDNA using the selected classifier I, which contained four selected genes (*RGS10*, *STSIA6*, *RUNX2,* and *VIM*), and the supporting classifier II, which contained 15 biomarkers that indicated excessive liver cell death and the increase in the sensitivity of the assay, were 94% and 89%, respectively [59].

Alternatively, Xu et al. developed a 10-methylation marker panel for cfDNA (*BMPR1A* (cg10428836), *PSD* (cg26668608), *ARHGAP25* (cg25754195), *KLF3* (cg05205842), *PLAC8* (cg11606215), *ATXN1* (cg24067911), Chr 6:170 (cg18196829), Chr 6:3 (cg23211949), *ATAD2* (cg17213048), and Chr 8:20 (cg25459300)), which effectively distinguished patients with HCC from individuals with a hepatitis B virus infection, hepatitis C virus infection, fatty liver, or healthy controls. The panel was used to construct a diagnostic prediction model. Additionally, a prognostic model based on an 8-methylation marker panel in combination with clinical and demographic characteristics was developed. Both panels were superior to the traditional tumor marker alpha-fetoprotein and previously discovered methylation biomarkers [39]. Hlady et al. created a CpG panel with five markers (cg04645914, cg06215569, cg23663760, cg13781744, and cg07610777), which successfully distinguished HCC patients from patients with cirrhosis. The probes which were selected directly from cfDNA outperformed probes from primary tissue samples when tested in cfDNA [43].

Recently, a novel tumor-specific high-throughput blood test, epiLiver, based on cfDNA methylation profiles, was developed for the detection of HCC. The test combines four different CpG sites (cg02012576, cg03768777, cg05739190, and cg24804544) and is specific for the detection of HCC combined with a biomarker that is significant for the liver tissue (cg1412693). The four HCC-specific biomarkers were sufficient to detect 98% of HCC samples (in 98% of tumor samples, at least one of the four selected CpG sites was methylated). A major drawback of the test is its lack of specificity for HCC versus other cancer types. With the addition of the liver-specific biomarker, the distinction of primary malignant liver tumors from liver metastases was possible. By combining biomarkers that detect cancer with biomarkers that identify TOO, patients with HCC can be accurately separated from healthy individuals or patients with chronic hepatitis B virus infection. This led to an 84.5% sensitivity and a 95% specificity [25].

Another marker that was considered for the diagnosis of HCC was 5 hmC. Li et al. identified cancer-associated 5 hmC signatures in cfDNA [68], while Song et al. investigated the utility of 5 hmC alterations in cfDNA to predict the cancer type and stage in HCC, lung, and pancreatic cancers. They also found HCC-specific changes in 5 hmC profiles that could be used to monitor HCC treatment, recurrence, and progression. Most importantly, they showed that 5 hmC signatures could successfully distinguish HCC from other primary and metastatic cancers [33]. Cai et al. developed a diagnostic model that could successfully distinguish early-stage HCC patients from healthy individuals [69].

#### 5.1.2. Cholangiocarcinoma

CCA is a heterogeneous group of malignancies characterized by biliary differentiation. It is the most common biliary malignancy and the second most common hepatic malignancy (after HCC). Based on the anatomic location, it is classified into three categories: intrahepatic, perihilar, and distal CCA [70]. Because CCA can assume any of the histologic patterns of adenocarcinoma, distinguishing primary CCA from metastatic adenocarcinoma is difficult and, in some cases, impossible based on histopathologic features alone [56]. Therefore, the need for new biomarkers is even greater. Since CCA is rarer than HCC, only a few studies have been performed to characterize the CCA epigenome. The number of studies focusing on methylation changes in the ctDNA of CCA patients is even fewer (Table 2). The first CCA methylation biomarkers were detected in biliary brush and tissue samples (e.g., *CDO1*, *CNRIP1*, *SEPT9* and *VIM*, *CDKN2*, *SOCS3*, *RASSF1A*, and *APC*) [70,71,72]. In tissue and plasma samples, the promoters of *SHOX2* and *SEPT9* were found to be frequently hypermethylated [73]. A 2019 study searched for novel cfDNA methylation biomarkers in the serum that could help in the differential diagnosis between CCA and other biliary tract diseases. Two novel methylation biomarkers, *OPCMl* and *HOXD9,* that successfully distinguish CCA from other biliary diseases were identified. Combining both markers led to a 62.5% sensitivity and 100% specificity [74]. Unfortunately, the study did not investigate the differentiation between CCA and other primary malignant liver tumors. When differentiating between HCC and CCA, we must take into account that in rare cases, the tumor may contain distinct elements of both HCC and CCA (combined hepatocellular and cholangiocarcinoma), which further complicates the differentiation between primary liver malignancies [56].

### 5.2. Most Common Liver Metastases

#### 5.2.1. Colorectal Cancer

CRC is the most common primary cancer that metastasizes into the liver [9]. This is most likely due to the abundant portal and arterial blood supply from the colon and rectum to the liver [3]. High genomic concordance has been observed between the genetic and epigenetic phenotype of primary CRC and liver metastases [77,78,79]. Ju et al. compared the epigenetic profiles of stages I-III CRC with metastatic stage IV CRC. An analysis of epigenetic evolution revealed different methylation profiles between CRC with and without metastases, as well as confirming that most methylation changes occurred before metastases [80]. Nevertheless, some degree of epigenetic divergence in CRC liver metastases from the primary tumor is expected, as shown by Orjuela et al., who examined differences in the DNA methylome of CRC and CRC liver metastases. They showed that hypermethylation was maintained in metastatic CRC compared to the primary tumor, whereas significant hypomethylation was observed in liver metastases compared to the primary tumor [78].

Additionally, tissue-specific DNA methylation biomarkers were identified in order to distinguish between CRC patients with and without liver metastases. They identified a colon-specific (exonic region of the SESN3 gene on chromosome 11) and a liver-specific DMR (exonic region of the PTK2B gene on chromosome 8). The selected CpG sites within the DMR of the target tissues were hypermethylated in the target tissue (i.e., colon or liver) but were hypomethylated (>50%) in other tissues and blood cells (<5%). This enabled the identification and quantification of the liver- and colon-derived cfDNA in the patients’ plasma using digital droplet PCR (ddPCR). The amount of colon-derived cfDNA was higher in CRC patients with and without liver metastases compared to the healthy controls. Similarly, the amount of liver-derived cfDNA was significantly increased in HCC patients compared to the healthy controls. In addition, a positive correlation between the absolute concentration of liver-derived cfDNA in HCC patients and tumor size was observed. This study demonstrates that DMR analysis and the detection of tissue-specific methylation patterns in cfDNA by ddPCR could be used to distinguish patients with and without metastases to other organs [81]. Table 3 provides an overview of the methylation biomarkers for CRC in liquid biopsy.

Another area of interest in CRC is the differences between tumors arising from the left-sided and right-sided colon. Left-sided tumors are more likely to metastasize to the liver [3]. Methylation-location-specific differences have also been identified in addition to mutations, miRNA, and differential enzyme activity. The increased hypermethylation of CpG islands in right-sided tumors and increased hypomethylation in open seas within left-sided tumors suggest that methylation could play an important role in the CRC metastatic pattern. These alterations may potentially represent an important predictive factor for the development of liver metastases in CRC patients [89]. 

#### 5.2.2. Pancreatic Cancer

Liver metastases occur in up to 80% of patients with metastatic pancreatic cancer, making the pancreas the second most common TOO among histologically confirmed liver metastases [9,90]. A few studies focused on metastatic pancreatic ductal adenocarcinoma (PDAC), the most frequently observed type of pancreatic cancer, and its blood-based biomarkers (Table 4).

Lehmann-Werman et al. demonstrated that the proportion of pancreas-derived DNA increased in the blood of patients with pancreatic cancer [91]. Lapin et al. demonstrated that a higher amount of cfDNA and shorter fragment sizes might have valuable predictive value for disease progression in patients with advanced pancreatic cancer. Six patients with locally advanced disease and fifty-five with metastases, most of whom had liver metastases, were included [92]. A 2015 systematic review showed that a larger gene panel was needed to achieve sufficient accuracy for the diagnosis of pancreatic cancer [93]. Later, Hendriksen et al. established a methylation panel for 28 genes that were found to be potential diagnostic biomarkers for pancreatic adenocarcinoma in cfDNA. The hypermethylation status of 19 gene promoters significantly distinguished patients with adenocarcinoma of the pancreas from the control group. Additionally, a predictive diagnostic model was developed (*BMP3*, *RASSF1A*, *BNC1*, *MEST*, *TFPI2*, *APC*, *SFRP1,* and *SFRP2*) that successfully diagnosed patients with pancreatic cancer (I-IV) with 76% sensitivity and 83% specificity [94].

**Table 4:** An overview of methylation biomarkers for PDAC in liquid biopsy. PDAC: pancreatic ductal adenocarcinoma; cfDNA: cell-free DNA; MSP: methylation-specific polymerase chain reaction; MethDet56: microarray-mediated methylation analysis of 56 fragments in each sample.

Method	Sample Type	Number of Samples	Type of Methylation	Genes and/or Genetic Location	References
Clinical validation (MSP)	Plasma cfDNA	PDAC (*n* = 95), chronic pancreatitis (*n* = 97), acute pancreatitis (*n* = 59), patients negative for PAAD (*n* = 27)	Hyper	Diagnostic panel: *BMP3*, *RASSF1A*, *BNC1*, *MEST*, *TFPI2*, *APC*, *SFRP1*, *SFRP2* Other: *BNC1*, *NPTX2*, *PENK*, *CDKN2A*, *RASSF1A*, *SFRP1*, *SARP*, *APC*, *BC1*, *CDKN2B*, *ESR1*, *MGMT*, *MLH1*, *RARB*	[94]
Clinical validation (MethDet56)	Plasma cfDNA	30 chronic pancreatitis, 30 patients with pancreatic cancer, and 30 healthy controls	Hyper and hypo	*CCND2*, *DAPK1*, *MLH1*, *MGMT*, *MUC2*, *MYOD1*, *CDKN2B*, *CDKN1C*, *PGK1*, *PGR*, *RARB*, *RB1*, *SYK*	[95]
Clinical validation (multiplexed array-mediated analysis)	Plasma cfDNA	30 PDAC patients and healthy controls	Hypo	*CCND2*, *SOCS1*, *THBS1*, *PLAU*, *VHL*	[96]
Clinical validation (real-time PCR)	Serum cfDNA	40 PDAC patients, 60 with chronic pancreatitis, and 5 with benign biliary stone diseases	Hyper	*NPTX2*	[97]
Review of the literature	cfDNA	/	Hyper and hypo	*CUX2*, *REG1A*, *ADAMTS1*, *BNC1*, *MLH1*, *PGR*, *SYK*, *CCND2*, *CDKN1C*	[98]

Alternatively, Yu et al. used the integrative analysis of DNA methylation and gene expression data to demonstrate that genes related to liver metastasis are mainly regulated at the epigenetic level [99]. While the recent systematic review focused on genes that underwent aberrant methylation at different stages of PDAC pathogenesis. The most promising genes for detecting PDAC with cfDNA at all stages of the disease were *CUX2*, *REG1A* (two unmethylated regions that successfully identified circulating exocrine cfDNA in the pancreas), *ADAMTS1*, *BNC1*, *MLH1*, *PGR*, *SYK*, *CCND2,* and *CDKN1C* [98].

Although little heterogeneity in driver mutations has been observed between primary and metastatic PDAC tumors, significant epigenetic reprogramming may occur during cancer progression [100]. Specifically, McDonald et al. found differences in epigenetic reprogramming between regional and distant metastasis, leading to differences in the anabolic glucose metabolism, which may enhance tumorigenic fitness during the evolution of distant metastasis [101].

#### 5.2.3. Lung Cancer

Lung cancer is the most common cancer in which liver metastases are present at the time of primary cancer diagnosis [6]. The two main types are small-cell lung cancer (SCLC) and NSCLC, among which SCLC is more commonly associated with liver metastases. Both types are difficult to diagnose in initiation phases and are characterized by the presence of liver metastases in 20% of SCLC and 13.4% of NSCLC patients at stage IV [102,103]. The majority of patients are diagnosed with advanced-stage disease, with 30–40% of NSCLC patients having metastatic disease at the time of diagnosis [103]. The main cause of lung cancer is tobacco consumption, which results in increased methylation and other epigenetic alterations that are common in smokers [104]. Smoke induces chronic inflammation, increases reactive oxygen species generation, and elevates DNMT1 expression [105,106]. Given this, it is not surprising that lung cancer has one of the best-studied cancer epigenetic landscapes in cfDNA (Table 5). 

The usefulness of methylation signatures in the ctDNA of lung cancer was demonstrated in the serum of 200 patients by identifying hypermethylated promoter regions of tumor suppressor genes (*MGMT*, *CDKN2A*, *INK4A*, *RASSF1A*, *DAPK,* and *RARB* [107]. A correlation between the tumor volume and the amount of ctDNA was observed. Moreover, ctDNA was detectable in all late-stage NSCLC cases [110]. The most commonly studied methylation biomarkers in ctDNA from lung cancer patients are *SHOX2*, *RASSF1A*, *RARB*, *LINE-1*, *PCDKN2A*, *MGMT*, *DAPK*, *APC,* and *DLEC1* [109]. Several combinations of differentially methylated gene promoter regions were found to be more effective than single gene promoters (*RASSF1A*/*RARB2*, *SHOX2*/*PTGER4*, *RTEL1*/*PCDHGB6*, *HOXD10*/*PAX9*/*PTPRN2*/*STAG3*, *APC*/*AIM1*/*CDH1*/*DCC*/*MGMT*/*RASSF1A*) in distinguishing lung cancer patients from noncancerous controls [109]. In clinical practice, many of these DMRs have the potential to predict disease progression, including in patients with stage IV lung cancer (*SHOX2*, *RARB*/*RASSF1A*, *RARB*, *RASSF1A*/*APC*, *DCLK1*, *BRMS1*, *SOX17*, *SFN*, *CHFR,* and *APC*/*RASSF1A*/*CDH13*/*CDKN2A*) [109].

#### 5.2.4. Gastric Cancer

Gastric cancer is the second leading cause of cancer-related deaths worldwide, as the majority of patients are diagnosed at an advanced stage [111]. Approximately 95% of gastric malignancies are adenocarcinomas, and nearly 40% of gastric cancer patients develop distant metastases during the course of their disease [112,113]. A study of the metastatic spread in the Swedish registry found that the most common metastatic site of gastric cancer was in the liver (48% of cases) [114].

Different methylation biomarkers proved to be useful for the non-invasive detection of gastric cancer in blood samples (Table 6). Many hypermethylated genes can be differentially detected in the plasma or serum from patient samples: *CDKN2A*, *CDKN2A/CDH1*, *CDH1*, *PCDKN2A/CDH1/RARB RUNX3*, *ZIC1*, *HOXD10*, *RUNX*, *RASSF1A*, *PCDH10*, *RPRM,* and *MLH1* [115]. Many other candidate methylation genes have also been discovered and require further investigation, including *ZIC1*, *RASSF10*, *RNF180*, *SFRP1*, *IRX1*, *CYP26B1/KCNA4*, *SLC19A3*, *FAM5C/MYLK*, *ATP4B*, *XAF1*, *SOX17*, *SPG20 FLNC/THBS1/UCHL1/DLEC1*, *OSR2/VAV3/PPFIA3,* and *TFPI2* [115].

To evaluate the overall specificity and sensitivity of blood-based methylation tests for the detection of gastric cancer, a meta-analysis of 32 studies, including 4172 patients with gastric cancer and 2098 controls, was performed [116]. The results showed a high overall specificity (97%) and modest sensitivity, while the latter could be improved with panels covering multiple DMRs (from 57% to 76%).

Few studies investigated the usefulness of methylation biomarkers for gastric cancer metastasis detection and prognostic assessment, and even fewer in liquid biopsy specimens. A panel of the DNA methylation biomarkers, *GFRA1* and *ZNF382,* was developed to assess the feasibility of predicting gastric cancer metastases in 188 patients’ formalin-fixed paraffin-embedded samples. The hypomethylation of these two independent predictors was associated with a higher risk of developing metastases. Gastric cancer metastases in patients without a spread to regional lymph nodes or distant metastases were predicted with a 61.5% sensitivity and a 70.1% specificity [117]. Fang et al. used genome-wide methylation analysis to identify three hypermethylated DNA promoter regions (*ADAM19*, *FLI1,* and *MSC*), which were validated in the tissue and plasma samples from 141 gastric patients. The hypermethylation of *FLI1* was also associated with advanced gastric cancer and the development of liver metastases in both tissue and plasma samples [118].

**Table 6:** An overview of methylation biomarkers for gastric cancer in liquid biopsy. cfDNA: cell-free DNA; MSP: methylation-specific polymerase chain reaction; CORD: Restriction digital PCR assay.

Method	Sample Type	Number of Samples	Type of Methylation	Genes and/or Genetic Location	References
Clinical validation (MSP)	Tissue and plasma	Tissues: 14 GCs and 42 controls; plasma: 36 GCs and 38 controls	Hyper	Panel: *ELMO1*, *ZNF569*, *C13orf18*	[119]
Clinical validation (MSP)	Tissue and serum	Tissues and plasma of 202 GC patients and their corresponding para-cancerous histological normal tissues; 88 serum healthy controls	Hyper	*XAF1*	[120]
Clinical validation (CORD)	Serum cfDNA	50 patients with early gastric cancer and 61 control individuals	Hyper	*RUNX3*	[121]
Clinical validation (genome-wide methylation analysis)	Tissue and plasma cfDNA	141 tissue and 106 plasma samples	Hyper	*ADAM19*, *FLI1*, *MSC*	[118]
Review of the literature	Serum or plasma	/	Hyper and hypo	*CDKN2A*, *CDKN2A/CDH1*, *CDH1*, *CDKN2A/CDH1/RARB*, *RUNX3*, *ZIC1*, *HOXD10*, *RUNX*, *RASSF1A*, *PCDH10*, *RPRM*, *MLH1*, *RASSF10*, *RNF180*, *SFRP1*, *IRX1*, *CYP26B1/KCNA4*, *SLC19A3*, *FAM5C/MYLK*, *ATP4B*, *XAF1*, *SOX17*, *SPG20*, *FLNC/THBS1/UCHL1/DLEC1OSR2/VAV3/PPFIA3*, *TFPI2*	[115]
Review of the literature	Plasma and/or serum cfDNA	/	Hyper and hypo	*PCDKN2A*, *CDH1*, *MGMT*, *RARB*, *RNF180*	[122]

#### 5.2.5. Breast Cancer

Breast cancer is the most common cancer in women [123], which metastasizes to various organs in 50% of patients with stage IV disease [124]. Approximately 15% of liver metastases originating from breast cancer [3]. The prognosis is poor, and the median survival of breast cancer patients with liver metastases ranges from three to fifteen months [124]. 

One of the first studies that proposed the use of epigenetic biomarkers in plasma to detect breast cancer compared four methylation biomarkers, *APC*, *GSTP1*, *RASSF1A,* and *RARB,* in primary breast cancer tissue and plasma samples. The hypermethylation of at least one of the selected genes resulted in a 62% sensitivity and an 87% specificity. Additionally, Liu et.al showed that the *FHIT* promoter of hypermethylation in the serum might be useful for the early diagnosis of ductal breast carcinoma [125]. In addition, the methylation status of *RARβ2* and *APC* genes in serum samples was superior to traditional tumor markers in the detection of breast cancer [126]. The hypermethylation of both genes was detected at all grades and stages (Table 7).

Another research group identified potential biomarkers for breast cancer detection from TCGA HumanMethylation450 BeadChip data. The new biomarker candidates *SPAG6*, *NKX2-6*, *PER1,* and *ITIH5* (panel SNiPER) were validated and tested in two independent serum cohorts, benign controls, and a plasma cohort [130]. The high methylation frequency of the included biomarkers confirmed blood-based breast cancer detection.

Another six-gene methylation panel (promoter regions of *SFN*, *CDKN2A*, *MLH1*, *HOXD13*, *PCDHGB7,* and *RASSF1A*) in cfDNA achieved an 82.4% sensitivity and a 78.1% specificity in tumors larger than 2 cm [128]. The direct comparison of DNA methylation biomarkers in 153 breast cancer patients between ctDNA, CTC, and paired FFPE primary tumor samples revealed consistent methylation aberrations in all three sample types [131]. *SOX17* promoter methylation status in CTCs and ctDNA was comparable in patients with early and metastatic breast cancer [131]. Another study demonstrated the usefulness of the targeted methylation sequencing of selected CpG sites in cfDNA for breast cancer detection and classification. Advanced breast cancer was successfully detected in 91.7% of cases and accurately classified in 72.7% of cases [49].

It should be emphasized that methylation patterns in cfDNA from breast cancer patients have also been evaluated as a potential tool for treatment monitoring. The decrease in the methylation of *PGR*, *MDGI*, *PAX5,* and *RARB* after surgical tumor removal and drug treatment suggested that the longitudinal monitoring of methylation in multiple promoters could be used to evaluate drug response and surgical success [132].

## 6. Conclusions and Future Directions

The poor differentiation between malignant liver tumors and the high incidence of CUP emphasizes the need to develop new tools for cancer detection and characterization. The poor prognosis and low overall survival rates of these patients highlight the need for accurate and early TOO determination. In malignant liver tumors, new technologies have enabled the methylation profiling of ctDNA in liquid biopsies, which could become a valuable complementary tool and provide additional information for clinical decision-making. In primary cancer, the identification of novel changes in methylation levels during carcinoma progression could serve as biomarkers for early cancer detection, diagnosis, and monitoring using minimally invasive screening tests. In secondary liver malignancies, they could contribute to successful TOO prediction, cancer characterization, and determination of primary cancer. Thus, they could greatly improve the treatment of late-stage cancer patients. For all this, they could facilitate treatment selection and optimization and potentially lead to the discovery of new drugs. Our review of the literature provides insight into the methylation changes in ctDNA from patients with malignant liver tumors and can serve as a starting point for further research.

## Figures and Tables

**Table 1:** An overview of methylation biomarkers for HCC in liquid biopsy. HCC: hepatocelular carcinoma; ctDNA: circulating tumor DNA; cfDNA: cell-free DNA; HM450k: Infinium HumanMethylation450k BeadChip Array; PCR: polymerase chain reaction; MCTA-Seq: methylated CpG tandems amplification and sequencing; TELQA: quantitative allele-specific real-time target and signal amplification assays; MSP: methylation-specific polymerase chain reaction; MSRE-qPCR: methylation-sensitive restriction enzymes-based quantitative PCR; MS-HRM: methylation-sensitive high-resolution melting.

Method	Sample Type	Number of Samples	Type of Methylation	Genes and/or Genetic Location	References
Bioinformatics analysis and clinical validation	Plasma ctDNA	1098 HCC patients and 835 normal controls	Hypo and hyper	Diagnostic panel: *BMPR1A*, *PSD*, *ARHGAP25*, *KLF3*, *PLAC8*, *ATXN1*, chr6:170, chr6:3, *ATAD2*, chr8:20 Prognostic prediction panel: *SH3PXD2A*, *C11orf9*, *PPFIA1*, chr17:78, *SERPINB5*, *NOTCH3*, *GRHL2*, *TMEM8B*	[39]
Clinical validation (HM450k)	Tissue and cfDNA	127 non-tumor and 415 HCC tissue samples; 37 non-tumor and 37 HCC cfDNA samples	Hypo and hyper	Diagnostic panel: chr19:51 (intragenic region), *ALX3*, *WINT3A*, chr1:42 (intragenic region), *GJB6*	[43]
Clinical validation (pyrosequencing)	Plasma cfDNA	237 HCC cases and 257 controls	Hyper	*TBX*	[57]
Clinical validation (multiplex PCR)	cfDNA	135 HCC and 302 control samples	Hyper	Detection panel: *HOXA1*, *EMX1*, *TSPYL5*	[58]
Clinical validation (MCTA-Seq)	Tissue and plasma cfDNA	151 tissue and plasma samples	Hyper	*RGS10*, *ST8SIA6*, *RUNX2*, *VIM*	[59]
Clinical validation (TELQA and MCTA-Seq)	Tissue and plasma cfDNA	74 HCC and 29 control tissue samples; 116 HCC, 81 cirrhotic controls and 98 healthy control plasma samples	Hyper	Detection panel: *HOXA1*, *EMX1*, *AK055957*, *ECE1*, *PFKP*, *CLEC11A* (normalized by *B3GALT6* level yielded)	[44]
Clinical validation (PCR)	Plasma cfDNA	289 patients	Hyper	*SEPT9*	[60]
Clinical validation	Tissue and plasma cfDNA	116 tissues and 326 plasma samples	Hyper	*SOCS3*	[61]
Clinical validation (MSP)	Serum cfDNA	80 HCC, 40 liver cirrhosis, 40 chronic hepatitis B, and 20 healthy controls	Hypo	*UBE2Q1*	[62]
Clinical validation (MSRE-qPCR)	Plasma cfDNA	72 patients with HCC, 37 benign live diseases, and 41 normal controls	Hyper	*APC*, *GSTP1*, *RASSF1A*, *SFRP1*	[63]
Clinical validation (MSP)	Tissue and plasma cfDNA	25 tissue and 130 plasma samples	Hyper	*CDKN2B*, *PCDKN2A*	[64]
Clinical validation (targeted massively parallel semiconductor sequencing)	Plasma cfDNA	84 HCC, 26 chronic liver patients, and 84 controls	Hyper	*FBLN1*, *VIM*	[65]
Clinical validation (MSP)	Tissue and plasma cfDNA	24 tissue and plasma samples	Hyper	*APC*, *FHIT*, *CDKN2B*, *PCDKN2A*, *CDH1*	[46]
Meta-analysis	144 eligible studies included	2044 HCC and 1371 normal serums samples	Hypo and hyper	*RASSF1A*, *PCDKN2A*, *CDH1*, *RUNX3*, *GSTP1*, *WIF1*	[45]
Meta-analysis	33 eligible studies included	4113 subjects	Hyper	*RASSF1A*	[66]
Review of the literature	Plasma or serum ctDNA	3442 HCC and 2696 controls	Hyper	*DBX2*, *THY1*, *TGR5*, *MT1M*, *MT1G*, *INK4A*, *VIM*, *FBLN1*, *RGS10*, *ST8SIA6*, *RUNX*, *SEPT9*	[67]

**Table 2:** An overview of methylation biomarkers for CCA in liquid biopsy. CCA: cholangiocarcinoma; cfDNA: cell-free DNA; MS-HRM: methylation-sensitive high-resolution melting; PCR: polymerase chain reaction.

Method	Sample Type	Number of Samples	Type of Methylation	Genes and/or Genetic Location	References
Clinical validation (MS-HRM)	Serum cfDNA	40 CCA and 40 controls	Hyper	*OPCML*, *HOXD9*	[74]
Clinical validation (Real-time PCR)	Tissue and plasma	Tissue: 71 tumors with pared normal samples Plasma: 20 CCA patients and 100 control patients	Hyper	*SHOX2*, *SEPT9*	[73]
Meta-analysis	Tissue and serum/plasma	/	Hyper	*SHOX2*, *SEPT9*, *OPCML*, *HOXD9*	[75]
Review of the literature	cfDNA	/	Hyper	*CDKN2*, *SOCS3*, *RASSF1A*, *APC*	[76]

**Table 3:** An overview of methylation biomarkers for CRC in liquid biopsy. CRC: colorectal cancer; cfDNA: cell-free DNA; MCTA-Seq: methylated CpG tandems amplification and sequencing; MSP: methylation-specific polymerase chain reaction.

Method	Sample Type	Number of Samples	Type of Methylation	Genes and/or Genetic Location	References
Clinical validation (MCTA-Seq)	Tissue and plasma cfDNA	Tissue: 66 samples Plasma: CRC (*n* = 147) and controls (*n* = 136)	Hyper	*EMBP1*, *KCNQ5*, *CHST11*, *APBB1IP*, *TJP2*, *SEPT9*, *IKZF1* Additional panel with 80 markers	[82]
Clinical validation (Quantitative MSP)	Plasma cfDNA	27 plasma samples	Hyper	*B4GALT1*	[83]
Clinical validation (MethyLight PCR)	Tissue and plasma cfDNA	21 plasma and 32 tissue biopsy samples	Hyper	*SFRP1*, *SFRP2*, *SDC2*, *PRIMA1*	[84]
Clinical validation (MethyLight PCR)	Plasma cfDNA	113 CRC patients and 87 controls	Hyper	*C9orf50*, *KCNQ5*, *CLIP4*	[85]
Systematic review	Blood, stool, urine, and tissue	51 studies included	Hyper and Hypo	Diagnostic panel: *APC*, *MGMT*, *RASSF2A*, *WIF-1* Other genes: *SEPT9*, *OSMR*, *BMP3*, *EFHD1*, *PPP1R3C*, *SFRP1*, *SFRP2*, *SPG20*	[86]
Review of the literature	Blood	/	Hyper and Hypo	*SEPT9*, *APC*, *CDKN2A/CDKN2A*, *MLH1*, *ALX4*, *TMEFF2*, *NGFR*, *FRP2*, *NEUROG1*, *TPEF/HPP1*, *RUNX3*, *HLTF*, *HPP1*, *DFNA5*	[87]
Review of the literature	Blood	/	Hyper	Diagnostic panel: *APC*, *MGMT*, *RASSF2A*, *WIF-1* Other genes: *SEPT9*, *BCAT1/IKZF1*, *AGBL4*, *FLI1*, *TWIST1*, *SFRP2*, *ITGA4*, *GATA5*	[88]

**Table 5:** An overview of methylation biomarkers for lung cancer in liquid biopsy. NSCLC: non-small cell lung cancer; SCLC: small-cell lung cancer; cfDNA: cell-free DNA; MS-PCR: methylation-specific polymerase chain reaction.

Cancer Type	Method	Sample Type	Number of Samples	Type of Methylation	Genes and/or Genetic Location	References
Lung cancer, SCLC, NSCLC	Clinical validation (MS-PCR)	Serum cfDNA	91 lung cancer patients, 9 with other malignant diseases, and 100 nonmalignant pulmonary diseases	Hyper	*MGMT*, *PCDKN2A*, *INK4A*, *RASSF1A*, *DAPK*, *RARB*	[107]
NSCLC	Review of the literature	Plasma or serum	/	Hyper and Hypo	*MGMT*, *PCDKN2A*, *DAPK*, *APC*, *CDH13*, *FHIT*, *RARB*, *RASSF1A*	[108]
Lung cancer, SCLC, NSCLC	Review of the literature	Plasma or serum	/	Hyper and Hypo	Diagnostic marker combinations: *RASSF1A/RARB*, *SHOX2/PTGER4*, *RTEL1/PCDHGB6*, *HOXD10/PAX9/PTPRN2/STAG3*, *APC/AIM1/CDH1/DCC/MGMT/RASSF1A* Diagnostic and prognostic markers: *SHOX2*, *RARB2/RASSF1A*, *RARB*, *RASSF1A/APC*, *DCLK1*, *BRMS1*, *SOX17*, *SFN*, *CHFR*, *APC/RASSF1A/CDH13/CDKN2A*	[109]
NSCLC	Review of the literature	Plasma or serum	/	Hyper	*CDKN2A*, *PAK3*, *NISCH*, *KIF1A*, *OGDHL*, *BRMS1*, *FHIT*, *CTSZ*, *CCNA1*, *NRCAM*, *LOX*, *MGMT*, *DOK1*, *SOX15*, *TCF21*, *DAPK*, *RAR*, *RASSF1*, *CYGB*, *MSX1*, *BNC1*, *CTSZ*, *CDKN2A*	[104]

**Table 7:** An overview of methylation biomarkers for breast cancer in liquid biopsy. cfDNA: cell-free DNA; MSP: methylation-specific polymerase chain reaction; MSRED: methylation-sensitive restriction enzyme digestion; BSP: bisulfite sequencing method; HRM: high-resolution melting; MethDet56: microarray-mediated methylation analysis of 56 fragments in each sample.

Method	Sample Type	Number of Samples	Type of Methylation	Genes and/or Genetic Location	References
Clinical validation (MSP)	Serum cfDNA	121 women breast cancer patients, 79 patients with benign breast diseases, and 66 healthy volunteers	Hyper	*APC*, *RARβ2*	[126]
Clinical validation (Quantitative MSP)	Plasma cfDNA	Women with breast cancer (*n* = 93) compared with control women (*n* = 76)	Hyper	*APC*, *GSTP1*, *RASSF1A*, *RARB2*	[127]
Clinical validation (MethyLight)	cfDNA in serum	49 cases including breast cancer patients, patients with benign breast diseases, and healthy women	Hyper	Panel: *SFN*, *CDKN2A*, *MLH1*, *HOXD13*, *PCDHGB7*, *RASSF1A*	[128]
Clinical validation (MSRED followed by Real-time PCR)	cfDNA in plasma	26 human BCs and 10 healthy controls	Hypo	*LINE-1*	[129]
Clinical validation (BSP and HRM)	Serum cfDNA	36 patients with invasive breast ductal carcinoma (BDC group), 30 patients with breast fibroadenoma (BFA group), and 30 healthy individuals	Hyper	*FHIT*	[125]
Clinical validation (Pyrosequencing)	Plasma and serum cfDNA	Serum test cohort (*n* = 103), a serum validation cohort (*n* = 368), and a plasma cohort (*n* = 125)	Hyper	Panel: *SPAG6*, *NKX2-6*, *PER1*, *ITIH5*	[130]
Clinical validation (MSP)	FFPE, whole blood (CTC), and plasma cfDNA	153 patients and 49 healthy controls	Hyper	*SOX17*	[131]
Clinical validation (MethDet-56)	Plasma cfDNA	20 patients and 20 healthy controls	Hyper	*RARB*, *ESR1*, *PGR*, *MDGI*, *MGMT*	[132]
Meta-Analysis	Tissue and blood	/	Hyper	*SNF*	[133]
Clinical validation (MSP)	Serum cfDNA	121 women breast cancer patients, 79 patients with benign breast diseases, and 66 healthy volunteers	Hyper	*APC*, *RARβ2*	[126]
Clinical validation (Quantitative MSP)	Plasma cfDNA	Women with breast cancer (*n* = 93) compared with control women (*n* = 76)	Hyper	*APC*, *GSTP1*, *RASSF1A*, *RARB2*	[127]
